# Multifunctional CaCO_3_@Cur@QTX125@HA nanoparticles for effectively inhibiting growth of colorectal cancer cells

**DOI:** 10.1186/s12951-023-02104-w

**Published:** 2023-09-29

**Authors:** Shengyun Hu, Kunkun Xia, Xiaobei Huang, Ye Zhao, Qingqing Zhang, Dongdong Huang, Weiyi Xu, Zhengju Chen, Chenfei Wang, Zhiyong Zhang

**Affiliations:** 1 Department of Colorectal and Anal Surgery, The First Affiliated Hospital of Zhengzhou University, Zhengzhou, 450052 China; 2 Department of Gastroenterology, The First Affiliated Hospital of Zhengzhou University, Zhengzhou, 450052 China; 3 Pooling Medical Research Institutes of 100Biotech, Beijing, 100006 China; 4 Department of Dermatology, National Children’s Medical Center, Children’s Hospital of Fudan University, Shanghai, 201102 China; 5 Chongqing Institute of Green and Intelligent Technology, Chinese Academy of Sciences, Chongqing, 400714 China

## Abstract

Colorectal cancer (CRC) is a major cause of cancer-related deaths in humans, and effective treatments are still needed in clinical practice. Despite significant developments in anticancer drugs and inhibitors, their poor stability, water solubility, and cellular membrane permeability limit their therapeutic efficacy. To address these issues, multifunctional CaCO_3_ nanoparticles loaded with Curcumin (Cur) and protein deacetylase (HDAC) inhibitor QTX125, and coated with hyaluronic acid (HA) (CaCO_3_@Cur@QTX125@HA), were prepared through a one-step gas diffusion strategy. Dynamic light scattering (DLS), transmission electron microscopy (TEM), and scanning electron microscopy (SEM) showed that CaCO_3_@Cur@QTX125@HA nanoparticles have uniform spherical morphology and elemental distribution, with diameters around 450 nm and a Zeta potential of − 8.11 mV. The controlled release of Cur from the nanoparticles was observed over time periods of 48 h. Cellular uptake showed that CaCO_3_@Cur@QTX125@HA nanoparticles were efficiently taken up by cancer cells and significantly inhibited their growth. Importantly, CaCO_3_@Cur@QTX125@HA nanoparticles showed specific inhibitory effects on CRC cell growth. Encouragingly, CaCO_3_@Cur@QTX125@HA nanoparticles successfully internalized into CRC patient-derived organoid (PDO) models and induced apoptosis of tumor cells. The multifunctional CaCO_3_@Cur@QTX125@HA nanoparticles hold promise for the treatment of CRC.

**Supplementary Information:**

The online version contains supplementary material available at 10.1186/s12951-023-02104-w.

## Introduction

Colorectal cancer (CRC) is a malignant tumor that poses a serious threat to human health, with high incidence and mortality rates worldwide [1–4]. The occurrence and development of CRC are influenced by various genetic and environmental factors, including age, family history, diet, obesity, smoking, alcohol consumption, and inflammatory bowel disease, among others [5, 6]. CRC not only impacts the quality of life for patients but also places a significant burden on society and the economy. Currently, the treatment of CRC mainly relies on traditional methods such as surgery, chemotherapy [7, 8], and radiotherapy [9], but these approaches have several limitations, including low efficacy, lack of specificity, and significant side effects [10]. Therefore, there is an urgent need to develop novel anti-cancer strategies.

In recent years, epigenetic regulation has gained widespread attention for its important role in tumorigenesis and tumor progression [11, 12]. Epigenetics refers to a series of molecular mechanisms that do not involve changes in DNA sequence but result in alterations in gene expression, including DNA methylation, histone modifications, and non-coding RNA regulation, among others [11]. Epigenetic regulation can affect various biological processes in tumor cells, such as proliferation, differentiation, migration, invasion, angiogenesis, apoptosis, and autophagy, thereby participating in tumorigenesis, development, and metastasis [13–16]. Histone deacetylase 6 (HDAC 6) is a specific deacetylase for tubulin, which is involved in regulating various processes in tumor cells, such as proliferation, migration, invasion, metabolism, and immune evasion [17–20]. HDAC 6 is overexpressed in multiple tumors and is associated with poor prognosis, especially in CRC [21, 22]. Therefore, HDAC 6 represents a promising anticancer target.

QTX125 is a novel HDAC 6-specific inhibitor with potent anticancer activity. It is a derivative of Tubacin, a selective HDAC 6 inhibitor discovered by Haggarty et al. [23]. QTX125 effectively inhibits the enzymatic activity of HDAC 6, leading to increased acetylation levels of tubulin and histones, resulting in cell cycle arrest, apoptosis, and autophagy in tumor cells. Moreover, QTX125 enhances the sensitivity of tumor cells to chemotherapy drugs by inhibiting HDAC 6-mediated ubiquitin–proteasome system and autophagy [24]. Montserrat et al. demonstrated significant antitumor effects of QTX125 both in vitro and in vivo, particularly in solid tumor cells with high drug resistance [25]. However, the efficacy of QTX125 alone in CRC treatment is limited, mainly due to its lack of selectivity, short half-life, and weak drug activity. Therefore, it is necessary to develop a safe and effective nano-carrier to deliver QTX125, in order to enhance CRC treatment.

In drug delivery, combination therapy is an effective strategy to improve therapeutic efficacy [26–29]. Curcumin (Cur) is a natural polyphenol derived from turmeric, which exhibits a wide range of biological activities, including antioxidant, anti-inflammatory, anti-angiogenic, and anticancer properties [30, 31]. Cur can induce apoptosis and autophagy in CRC cells through various mechanisms, while also enhancing the immune system's ability to clear tumors. Cur inhibits multiple signaling pathways, such as NF-κB, AP-1, STAT3, Wnt/β-catenin, MAPK, PI3K/Akt, etc., thereby regulating downstream gene expression and protein activity [32, 33]. Giommarelli et al. studied the synergistic effect of combining Cur with HDAC inhibitors, and observed that it promoted tumor cell apoptosis [34]. However, Cur is often poorly absorbed in the gastrointestinal tract, leading to low bioavailability. Therefore, the simultaneous delivery of Cur and QTX125 using nano-carriers is expected to significantly enhance CRC treatment efficacy.

**Scheme 1:**
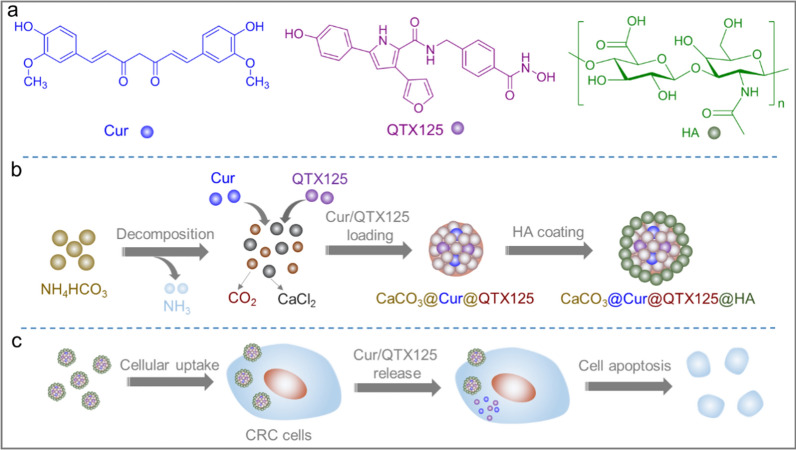
CaCO_3_ nanoparticles loaded with Cur/QTX125 and coated with HA can effectively inhibit the growth of CRC cells. **a** Chemical structures of Cur, QTX125 and HA; **b** The preparation of CaCO_3_@Cur@QTX125@HA nanoparticles. NH_4_HCO_3_ was first decomposed into NH_3_ and CO_2_, which diffused into the CaCl_2_ solution containing Cur and QTX125. Then, CO_2_ reacted with Ca^2+^ to form Cur and QTX125 loaded CaCO_3_@Cur@QTX125 nanoparticles, which were subsequently coated with HA to generate CaCO_3_@Cur@QTX125@HA nanoparticles; **c** CaCO_3_@Cur@QTX125@HA nanoparticles can be taken up by CRC cells, Cur and QTX125 can be released to induce cell apoptosis

In this study, We prepared amorphous and uniform CaCO_3_ nanoparticles using a one-step gas diffusion strategy and successfully encapsulated Cur and QTX125 within them (Scheme 1). CaCO_3_ nanoparticles possess excellent biocompatibility, biodegradability, and pH sensitivity, making them ideal drug carriers. In order to achieve a synergistic anticancer effect, we have designed a drug delivery system based on amorphous calcium carbonate (CaCO_3_) nanoparticles to co-deliver Curcumin (Cur) and QTX125. To enhance cellular uptake, we modified the surface of the nanoparticles with hyaluronic acid (HA), which can bind to CD44 receptors over-expressed on the surface of CRC cells, thereby enhancing the targeting and endocytosis of the nanoparticles. The nanoparticles were characterized with scanning electron microscopy (SEM), transmission electron microscopy (TEM), Fourier-transform infrared spectra (FTIR), X-ray diffraction (XRD) and Zetasizer. Release of Cur from the nanoparticles was determined. Cellular uptake of the nanoparticles and their inhibitory effects on the growth of cancer cells were investigated. Furthermore, the efficacy of the nanoparticles on induced apoptosis of tumor cells in PDO models was verified.

## Experimental

### Materials

Calcium chloride (CaCl_2_, 98%), ammonium bicarbonate (NH_4_HCO_3_, 99%), Cur (C_21_H_20_O_6_, 98%), hyaluronic acid (HA, molecular weight = 97.0%), and ethanol (99.5%) were purchased from Shanghai Aladdin Bio-Chem Technology Co., Ltd. QTX125 (98%) was purchased from MedChemExpress, while Rhodamine B (C_28_H_31_ClN_2_O_3_, 98%), Paraformaldehyde (PFA), Triton X-100, Type II collagenase, growth factor reducing matrix gel, matrix adhesive were purchased from Sigma-Aldrich. 4′,6-diamidino-2-phenylindole (DAPI, C_16_H_15_N_5_·2HCl, 98%), Hoechst33342 (C_27_H_28_N_6_O, 97%) was obtained from Solarbio and alamarBlue (C_12_H_6_NNaO_4_, 75%) was obtained from Yeasen Biotechnology and used in accordance with the manufacturers’ protocols. Phosphate-buffered saline (PBS, pH = 7.2–7.4, 0.01 M), sodium acetate buffer (pH = 5.2, 3 M), and Hanks' Balanced Salt Solution (HBSS) were purchased from Beyotime Biotechnology. Dulbecco's Modified Eagle Medium (DMEM), Minimum Essential Medium (MEM), Roswell Park Memorial Institute (RPMI) 1640 Medium, F12 medium, fetal bovine serum (FBS), and penicillin/streptomycin (P/S) were purchased from Thermo Fisher Scientific. Growth factors Responsin 1 (120–38), EGF (100–15), and Wnt-3a (315–20), primary rabbit monoclonal antibody CEA, CA19-9 and AlexaFluor® 488-conjugated goat anti-rabbit secondary antibody were purchased from Abcam. B12 (17504044) was purchased from Life Technology. Nicotinamide (N0636), N-acetylcysteine, and SB202190 (S7076) were obtained from Sigma. All reagents and materials used in the experiment were of analytical grade.

### Preparation of CaCO_3_ nanoparticles

CaCO_3_ nanoparticles were synthesized using a gas diffusion method [35, 36]. Briefly, 150 mg of CaCl_2_ was dissolved in 100 mL of ethanol in a beaker and stirred for 20 h. Next, 5 g of NH_4_HCO_3_ was placed outside the beaker. The entire setup was then sealed and kept at 30 °C while stirring overnight. After that, the dispersed CaCO_3_ nanoparticles were collected and washed three times with deionized water through centrifugation (6000 rpm, 20 min). The final product was lyophilized and then redispersed in deionized water at a concentration of 100 mg/mL.

### Preparation of CaCO_3_@Cur@QTX125 nanoparticles

CaCO_3_@Cur@QTX125 nanoparticles were synthesized using the gas diffusion method, which is similar to the process used for the synthesis of CaCO_3_ nanoparticles. Firstly, 5 mg of QTX125, 5 mg of Cur, and 150 mg of CaCl_2_ were dissolved in 100 mL of ethanol and stirred for 20 h until fully dissolved. Then, 5 g of NH_4_HCO_3_ was placed outside the beaker containing the mixture solution. The entire setup was then sealed and kept at 30 °C while stirring overnight. The resulting nanoparticles were collected and washed three times with deionized water through centrifugation (6000 rpm, 20 min), and finally lyophilized before being redispersed in deionized water.

### Preparation of CaCO_3_@Cur@QTX125@HA nanoparticles

To prepare CaCO_3_@Cur@QTX125@HA nanoparticles, 1 mg of the previously prepared CaCO_3_@Cur@QTX125 nanoparticles was dispersed in 10 mL of deionized water. Then, 1 mg of HA was added to the solution and left stirring overnight. The mixture was subsequently centrifuged and the resulting CaCO_3_@Cur@QTX125@HA nanoparticles were collected. They were then washed three times with deionized water via centrifugation and finally obtained through freeze-drying.

### Preparation of rhodamine B-labeled CaCO_3_@Cur@QTX125@HA nanoparticles

To prepare Rhodamine B-labeled CaCO_3_@Cur@QTX125@HA nanoparticles, we followed the same procedure as described above, except that Rhodamine B (RhB) was added to the ethanol solution containing Ca^2+^ ions, Cur and QTX125. The concentration of RhB was 0.1 mg/mL. The obtained RhB-labeled nanoparticles were washed with PBS and collected by centrifugation. The fluorescence intensity of the nanoparticles was measured by a fluorescence spectrophotometer. The results showed that the nanoparticles exhibited strong fluorescence emission at 580 nm when excited at 550 nm, indicating the successful labeling of RhB.

### Characterization of nanoparticle compositions, crystal structures and morphologies

The chemical compositions of CaCO_3_, CaCO_3_@Cur@QTX125, and CaCO_3_@Cur@QTX125@HA nanoparticles were determined using an attenuated total reflectance Fourier-Transform Infrared spectrometry (ATR-FTIR, Nicolet iS50) in the 4000–400 cm^−1^ range. The crystal structures of the nanoparticles were identified through powder X-ray diffraction (XRD) (XRD-6100) using Cu Kα radiation. Morphologies and energy-dispersive spectroscopy (EDS) maps of the nanoparticles were imaged with scanning electron microscopy (SEM, MAIA3 LMH) and transmission electron microscopy (TEM, Thermo Fisher Scientific, Talos L120C G2) at 120 kV. To prepare TEM samples, 10 mg of CaCO_3_, CaCO_3_@Cur@QTX125, and CaCO_3_@Cur@QTX125@HA nanoparticles were suspended in 10 ml of deionized water and vortexed completely. Then, 5 μl of the nanoparticle suspensions were dropped onto carbon support films, quickly frozen dried, and imaged.

### Measurements of nanoparticle sizes and Zeta potentials by dynamic light scattering (DLS)

CaCO_3_@Cur@QTX125@HA nanoparticles were suspended in 1.5 mL of deionized water and thoroughly vortexed. The size and zeta potential of the nanoparticles were measured using a Malvern Instruments Zetasizer (Nano ZSE, He–Ne laser, λ = 632 nm) at a scattering angle of 173° and a temperature of 25 ºC. All measurements were repeated at least three times.

### Measurements of pH-dependent release of Cur from CaCO_3_@Cur@QTX125 and CaCO_3_@Cur@QTX125@HA nanoparticles

A Cur standard curve was first plotted at the Cur concentration of 5, 10, 20, 40 and 80 μg/ml. To determine the pH-dependent release profiles of Cur, 500 mg of CaCO_3_@Cur@QTX125 or CaCO_3_@Cur@QTX125@HA nanoparticles were suspended in 15 mL of PBS (pH 7.2–7.4, 0.01 M) or sodium acetate buffer (pH 5.2, 3 M), vortexed, and then kept under magnetic stirring at 25 ºC. At different time intervals, 1 mL of the solution was extracted and centrifuged, the concentration of Cur in the supernatant was measured using a microplate reader (Synergy Hybrid H1, Biotek) at 425 nm. All measurements were repeated at least three times.

### Cellular experiments

#### Cell culture

Human cervical cancer cells (HeLa, ATCC), human colon cancer cell (HCT116, ATCC), HCV-29 bladder epithelial cells (HCV-29, ATCC) and intestinal epithelioid cells (IEC-6, ATCC) were cultured in DMEM supplemented with 1% P/S and 10% FBS. Human bladder transitional carcinoma cells (UC-3 cells, ATCC) were cultured in MEM supplemented with 1% P/S and 10% FBS, while human colorectal cancer cells (HT-29, ATCC) were cultured in RPMI1640 medium supplemented with 1% P/S and 10% FBS. All cells were incubated in a humidified incubator under standard culture conditions of 37 °C and 5% CO_2_.

#### Cellular uptake of CaCO_3_@Cur@QTX125@HA nanoparticles

The CaCO_3_@Cur@QTX125@HA nanoparticles were first labeled with Rhodamine B and then suspended in PBS (Contained 5% DMSO) at a concentration of 40 μg/ml. In a 96-well plate, HCT116 cells, HT29 cells, HCV-29 cells and UC-3 cells were seeded at a density of 5000 cells/well in 100 μl cell culture medium and cultured overnight. 10 μl of Rhodamine B-labeled CaCO_3_@Cur@QTX125@HA nanoparticles were diluted with 90 μl of cell culture medium containing 10% FBS. The medium was removed from the cell culture plate and replaced with 200 μl of the Rhodamine B-labeled CaCO_3_@Cur@QTX125@HA nanoparticles. After 6 h, the medium was removed and the cells were washed three times with PBS. The cells were then stained with Hoechst33342 and visualized using a fluorescence microscope (CK53, Olympus).

#### In vitro cell metabolic activity evaluation

Cell metabolic activity was measured using alamarBlue assays according to the manufacturer's protocol. HCT116 cells, HT29 cells, HCV-29 cells, UC-3 cells, HeLa cells, and IEC-6 cells were seeded at a density of 1 × 10^4^ cells per well in 100 μl of cell culture medium and cultured until 80% confluency in a 96-well plate. The CaCO_3_@Cur@QTX125@HA nanoparticles were suspended in PBS at various concentrations (6.25, 12.5, 25, 50, 100, 200 and 400 μg/mL). Next, the cell culture medium was removed from the plate, and 100 μl of the CaCO_3_@Cur@QTX125@HA nanoparticles at various concentrations was added to each well. After 24 or 48 h of incubation, the medium was removed, and the cells were washed twice with HBSS. Next, 100 μL of a 10% diluted alamarBlue solution in DMEM, MEM, or RPMI1640 was added to each well, and the cells were incubated for an additional 0.5–1 h. Finally, the fluorescence intensity of the supernatant was measured at 530 nm excitation and 590 nm emission using a microplate reader (Synergy Hybrid H1, Biotek). Cells that were not treated with nanoparticles were used as controls to define 100% viability.

#### CRC organoid experiment

##### Patient derived organoid (PDO) model

CRC tumor samples were collected from the Colorectal and Anorectal Surgery Department of the First Affiliated Hospital of Zhengzhou University. This experiment was approved by Research and Clinical Experiment Ethics Committee of the First Affiliated Hospital of Zhengzhou University (NO. 2023-KY-0484). To construct a patient-induced organoids model of CRC, fresh tumor tissue obtained during surgery was immediately transported to DMEM/F12 medium supplemented with 1% P/S. The tissue was cut into approximately 2 mm slices and then digested with a digestion solution (DMEM/F12 containing 2.5 mg/mL type II collagenase) at 37 °C for 1 h. After centrifugation, the supernatant was removed, and 1 ml of PBS was added to resuspend the organoid precipitate. Then, 9 ml of PBS was added, and the mixture was centrifuged at 400 g for 5 min before being mixed with the growth factor-reducing matrix gel. The mixture was distributed into 24-well plates, with each well containing approximately 50,000 cells and 25 μL of matrix adhesive. Once the matrix gel was solidified, 500 μL of culture medium was added. The culture medium was replaced every 2–3 days for approximately 2 weeks, during which the growth and morphological changes of similar organs were observed. Photos were taken on days 1, 3, 5, 9, and 12, respectively. The culture medium was composed of advanced DMEM/F12, 1% P/S, 1X B27, nicotinamide, N-acetylcysteine, SB202190, and growth factors Responsin, EGF and Wnt-3a. After organoid generation, different concentrations of CaCO_3_, CaCO_3_@Cur@QTX125, and CaCO_3_@Cur@QTX125@HA nanoparticles were added to the culture medium, and the medium was replaced every 3 days.

##### Internalization of nanoparticles in PDO models

The PDO models were first implanted into petri dishes. Next, 200 μl of Rhodamine B-labeled CaCO_3_@Cur@QTX125@HA nanoparticles (50 μg/mL) suspended in PBS (contained 5% DMSO) were added to the petri dishes and incubated for 24 h. The cells were then fixed with 4% paraformaldehyde for 20 min, washed three times with PBS, and stained with DAPI. After staining, the cells were washed again with PBS before being sealed with climbing sheets. The uptake of CaCO_3_@Cur@QTX125 @HA nanoparticles in the PDO models was visualized using a confocal microscopy (ANDOR Dragonfly, UK) with a Z-stack model.

##### TUNEL (EdU TUNEL) imaging

To determine cell apoptosis in the PDO tissues, TUNEL (Terminal deoxynucleotidyl transferase dUTP nick end labeling) imaging was performed using the TUNEL Alexa Fluor imaging Assay (Invitrogen), following the manufacturer's instructions. Cryofixed graft tissue sections were fixed in 4% PFA for 15 min at room temperature and then permeabilized in 0.25% Triton X-100 in PBS for 20 min at room temperature. The sections were then incubated at 37 °C with a labeled reaction mixture containing 4.0 μL (TdT) and a nucleotide mixture including 1.0 μL (FITC-12-dUTP) for 1 h under dark conditions. After washing three times, they were counterstained with DAPI. Apoptosis was determined by laser confocal microscopy (LEICA SP8, Germany).

##### Immunofluorescence

PDOs were seeded onto 96-well plates and treated with Rhodamine B-labeled CaCO_3_, CaCO_3_@Cur@QTX125, and CaCO_3_@Cur@QTX125@HA nanoparticles for 24 h. After fixation with 4% PFA for 30 min, the PDOs were permeabilized with PBST (PBS containing 0.5% Triton X-100) for 5 min. The PDOs were then incubated overnight at 4 °C with primary antibodies CEA (1:200) and CA19-9 (1:200). After washing with PBST 3 times, the samples were further incubated with AlexaFluor^®^ 488-conjugated goat anti-rabbit secondary antibody (1:200). Nuclei were stained with DAPI, and the images were captured using a confocal microscope (LEICA SP8, Germany).

### Statistical analysis

The cell viability was analyzed using Student’s *t*-test, and the results are presented as mean ± SD (standard deviation) values. Mean and SD were calculated from at least three independent experimental replications. A *p* value less than 0.05 was considered statistically significant.

## Result and discussion

### Preparation of various nanoparticles

**Fig. 1:**
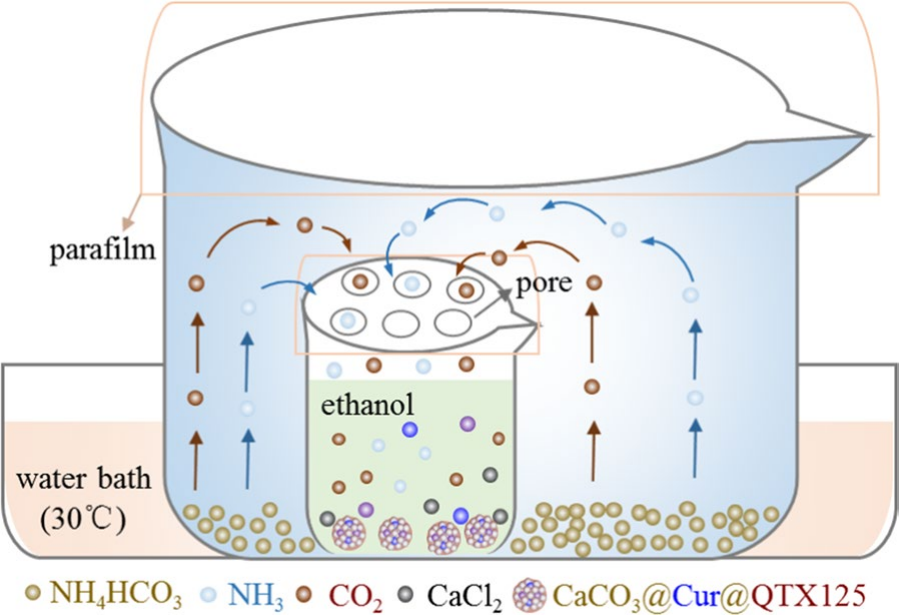
Schematic illustration of the gas diffusion device used for the preparation of CaCO_3_@Cur@QTX125 nanoparticles

To synthesize CaCO_3_ nanoparticles and co-encapsulate Curcumin (Cur) and QTX125 within them, we employed a one-step gas diffusion method. This method allows the simple chemical reaction at room temperature to generate amorphous CaCO_3_ nanoparticles and simultaneously encapsulate the drugs in one step. Furthermore, we surface-modified the nanoparticles with hyaluronic acid (HA) to enhance their targeting and uptake by CRC cells. Various characterization techniques were used to assess the morphology, size, charge, composition, and structure of the nanoparticles. As shown in Fig. 1, the process of preparing CaCO_3_ and CaCO_3_@Cur@QTX125 nanoparticles is as follows: In a sealed system, NH_4_HCO_3_ decomposes into CO_2_ and NH_3_ (Eq. 1), which then diffuses into an ethanol solution containing Ca^2+^ ions. Under alkaline conditions, the carbonate reacts with Ca^2+^ ions to form CaCO_3_ nanoparticles (Eqs. 2, 3).

1 $$ {\text{NH}}_{{4}} {\text{HCO}}_{3} \to {\text{NH}}_{{3}} + {\text{CO}}_{2} + {\text{H}}_{2} {\text{O}} $$

2 $$ {\text{CaCl}}_{{2}} \to {\text{Ca}}^{2 + } + 2\,{\text{Cl}}^{ - } $$

3 $$ {\text{Ca}}^{{2 + }} {\text{ + CO}}_{{3}}^{{2 - }} \to {\text{CaCO}}_{{3}} $$

**Fig. 2:**
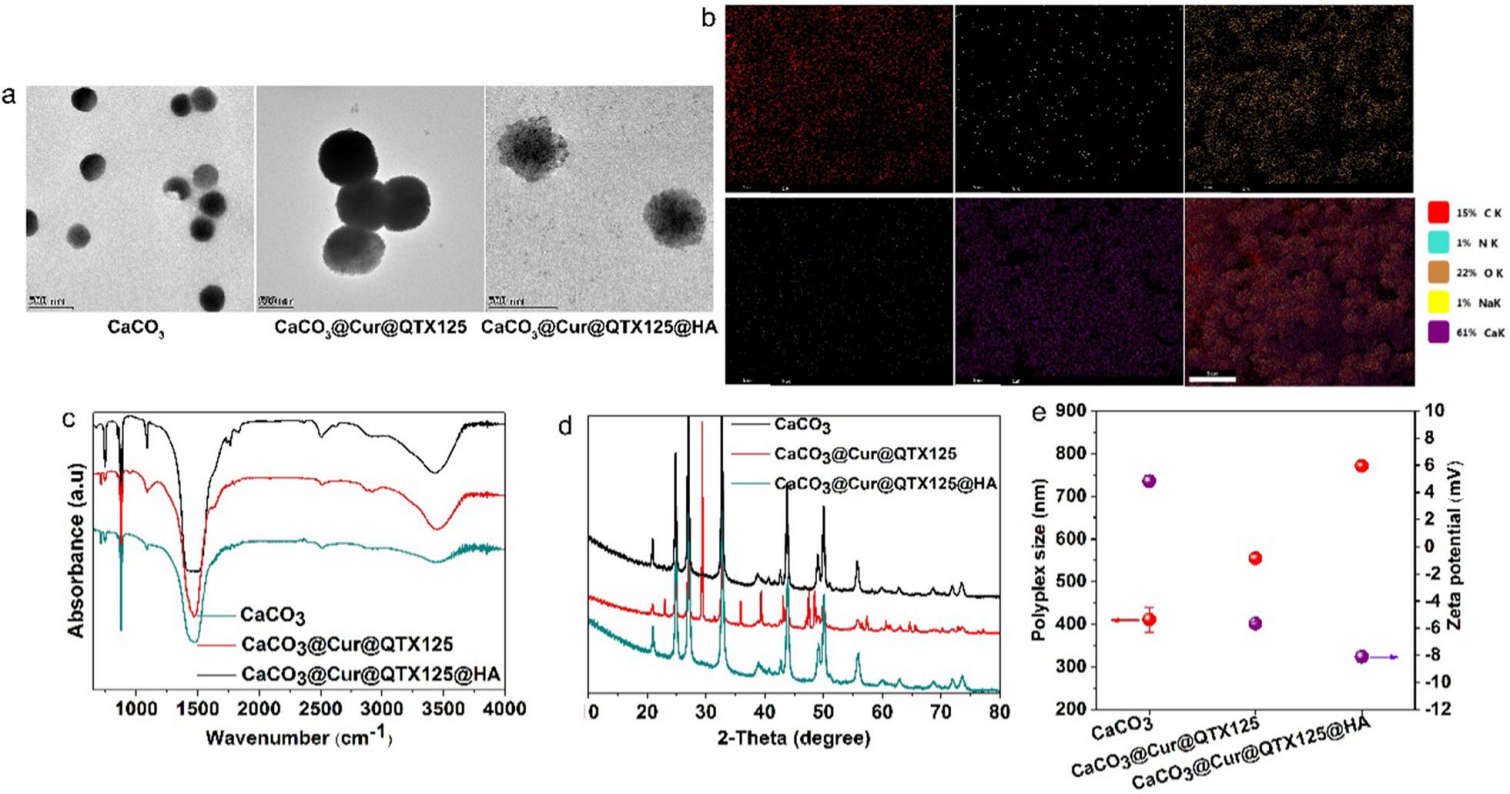
**a** Representative TEM images of CaCO_3_, CaCO_3_@Cur@QTX125 and CaCO_3_@Cur@QTX125@HA nanoparticles, scale bars represent 500 nm. **b** Elemental mapping of CaCO_3_@Cur@QTX125@HA nanoparticles, scale bars represent 5 μm. **c** FTIR spectra of CaCO_3_, CaCO_3_@Cur@QTX125 and CaCO_3_@Cur@QTX125@HA nanoparticles. **d** XRD patterns of CaCO_3_, CaCO_3_@Cur@QTX125 and CaCO_3_@Cur@QTX125@HA nanoparticles. **e** The size and Zeta potential of CaCO_3_, CaCO_3_@Cur@QTX125 and CaCO_3_ @Cur@QTX125@HA nanoparticles

To prepare CaCO_3_@Cur@QTX125 nanoparticles, Cur and QTX125 are dissolved in the ethanol solution containing Ca^2+^ ions. As CO_2_ and NH_3_ diffuse and react with Ca^2+^ ions to form CaCO_3_ nanoparticles, Cur and QTX125 are simultaneously encapsulated. To further prepare CaCO_3_@Cur@QTX125@HA nanoparticles, CaCO_3_@Cur@QTX125 nanoparticles are incubated with hyaluronic acid (HA). After centrifugation and washing with PBS, CaCO_3_, CaCO_3_@Cur@QTX125, and CaCO_3_@Cur@QTX125@HA nanoparticles are obtained (Additional file 1: Figures S1, S2, S3). SEM and TEM observations show that all three types of nanoparticles have uniform, spherical, and graded morphology, with diameters of approximately 250, 500 and 500, respectively (Fig. 2a). The size of CaCO_3_@Cur@QTX125 nanoparticles is larger than that of corresponding CaCO_3_ nanoparticles, consistent with DLS measurement results (Fig. 2e). After HA coating, the size of the nanoparticles further increases. Additionally, the Zeta potential of the nanoparticles decreases from 4.83 mV to − 5.66 mV, and further to − 8.11 mV, which helps prevent serum protein adsorption (Fig. 2e). Element mapping shows uniform distribution of carbon, nitrogen, oxygen, and calcium in CaCO_3_@Cur@QTX125@HA nanoparticles (Fig. 2b). FT-IR spectra indicate the appearance of asymmetric stretching split peaks of C-O at 1491 cm^−1^ and 1419 cm^−1^, confirming the uniform loading of Cur and QTX125 into the nanoparticles (Fig. 2c). The characteristic peak of the carbohydrate group appears at 1038 cm-1, confirming the successful coating of HA (Fig. 2c). XRD spectra show that CaCO_3_@Cur@QTX125 and CaCO_3_@Cur@QTX125@HA nanoparticles have similar amorphous structures (Fig. 2d), indicating that the loading of Cur and QTX125, as well as the HA coating, did not affect the crystallinity of the nanoparticles.

### Release of Cur from CaCO_3_@Cur@QTX125 and CaCO_3_@Cur@QTX125@HA nanoparticles

**Fig. 3:**
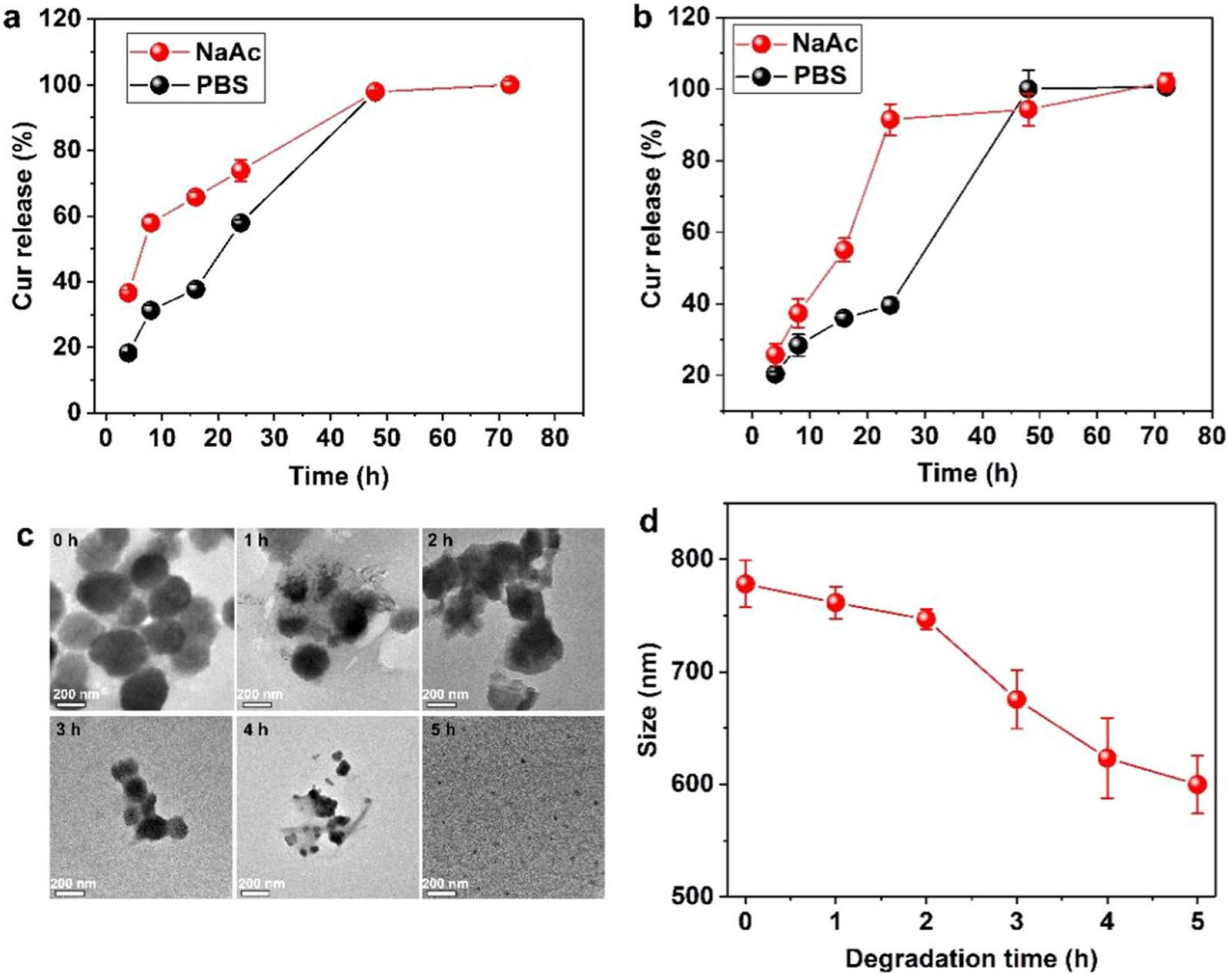
**a** The release profiles of Cur from CaCO_3_@Cur@QTX125 nanoparticles in PBS at a pH = 7.4 and NaAc at pH = 5.2. **a** The release profiles of Cur from CaCO_3_@Cur@ QTX125@HA nanoparticles in PBS at pH = 7.4 and NaAc at pH = 5.2. **c** Representative TEM images of CaCO_3_@Cur@QTX125@HA nanoparticles incubated in NaAc at a pH of 5.2 over different periods of time, scale bars represent 200 nm. **d** Time-dependent size changes of CaCO_3_@Cur@QTX125@HA in NaAc at a pH of 5.2 for different periods of time

Effective prolonged therapy for tumors requires the controlled release of therapeutics in tumor tissue. The release of Cur from CaCO_3_@Cur@QTX125 and CaCO_3_@Cur@QTX125@HA nanoparticles was first measured in PBS with a pH of 7.4. The results showed that at the early stage, around 18% of Cur was released from CaCO_3_@Cur@QTX125 nanoparticles (Fig. 3a). With incubation time increased to 8, 16, and 24 h, the accumulated release of Cur significantly increased and reached 31%, 37%, and 57%, respectively, but still less than 60%. However, after an increased incubation time of 48 h, more than 90% of the Cur was released from the nanoparticles, with almost all of it being released after XX hours. In contrast, the release of Cur in NaAc buffer with a pH of 5.2 was faster in the early stages than in PBS. For instance, after incubation for 4, 8, 16, and 24 h, the accumulated release of Cur was 36%, 57%, 65%, and 73%, which is 50%, 57%, 43%, and 22% higher than that in the PBS. However, after 48 and 72 h of incubation, similar amounts of Cur were released from CaCO_3_@Cur@QTX125 as in PBS. The relatively faster release of Cur in acidic solution can be attributed to the greater solubility of CaCO_3_ nanoparticles. After HA coating, the release of Cur from CaCO_3_@Cur@QTX125@HA nanoparticles during the first 24 h in both PBS and sodium acetate buffer was markedly reduced (Fig. 3b). For example, in PBS, after 4, 8, and 16 h of incubation, the accumulated release of Cur was 20%, 28%, and 35%, respectively. Similar to CaCO_3_@Cur@QTX125, the release of Cur from CaCO_3_@Cur@QTX125@HA nanoparticles in the acidic NaAc buffer was still relatively faster. These findings indicate that HA coating provides some protection to CaCO_3_, thereby prolonging the release of Cur.

To further elucidate the mechanisms underlying the release of Cur from the nanoparticles, CaCO_3_@Cur@QTX125@HA nanoparticles were observed with TEM after incubation for different periods in NaAc buffer. Initially, the CaCO_3_@Cur@QTX125@HA nanoparticles exhibited a uniform, spherical, and hierarchical morphology (Fig. 3c). After 1 h of incubation, some of the nanoparticles partially dissolved, resulting in a decrease in size from around 300 nm to less than 20 nm, and the nanoparticles presented an irregular morphology. With an increase in incubation time to 2, 3, and 4 h, the changes in size and morphology of the CaCO_3_@Cur@QTX125@HA nanoparticles became more noticeable. After 5 h of incubation, all the original CaCO_3_@Cur@QTX125@HA nanoparticles decomposed, and much smaller nanoparticles with a size of less than 40 nm were observed. Furthermore, DLS measurements confirmed the decrease in nanoparticle size with an increase in incubation time (Fig. 3d). Our results demonstrate that the release of Cur from the CaCO_3_@Cur@QTX125@HA nanoparticles was due to their slow degradation.

### Cellular uptake of CaCO_3_@Cur@QTX125@HA nanoparticles

**Fig. 4:**
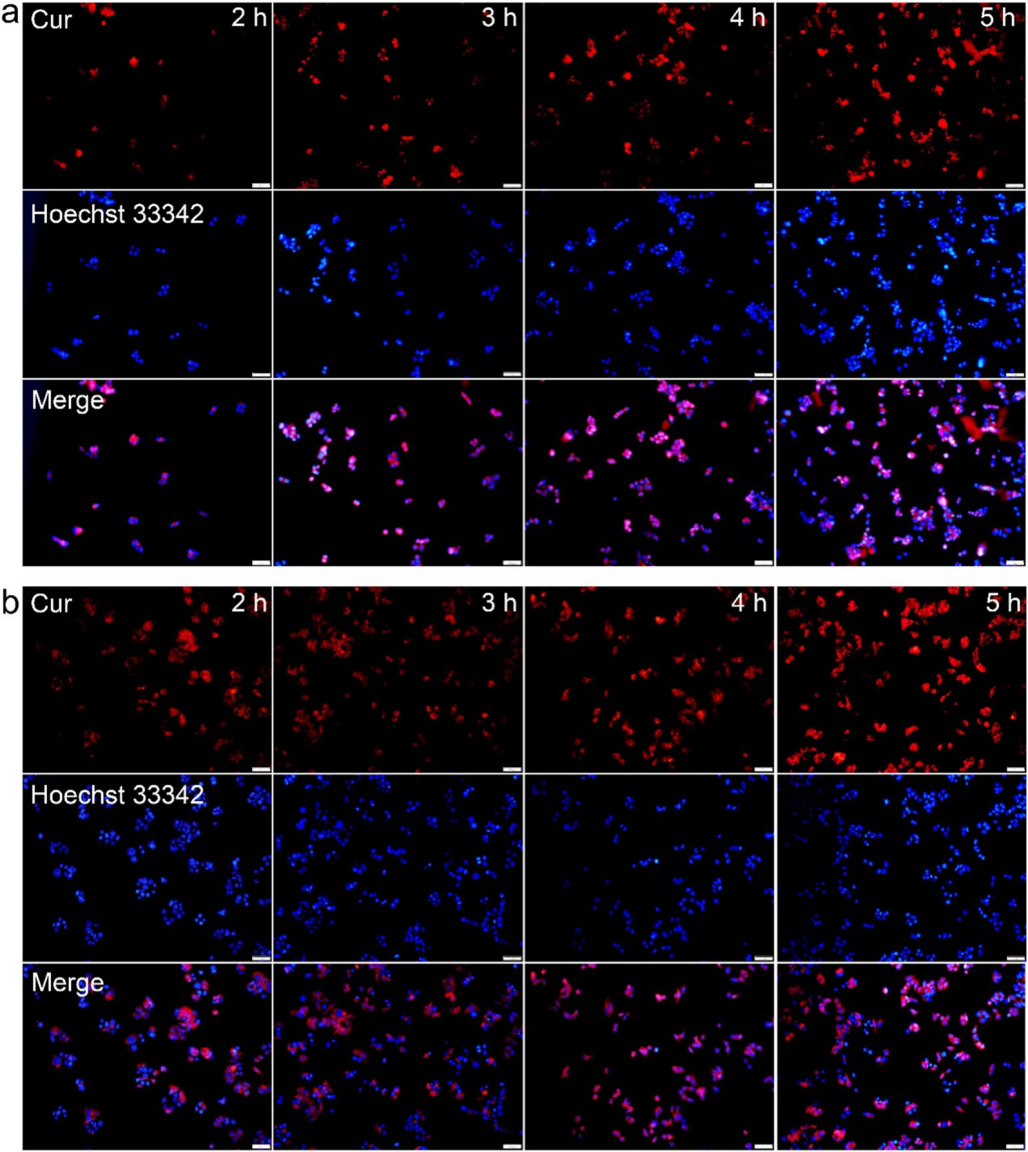
**a** Fluorescence images of HCT116 cells after incubation with CaCO_3_@Cur@ QTX125@HA for 2, 3, 4, and 5 h. **b** Fluorescence images of HT29 cells after incubation with CaCO_3_@Cur@QTX125@HA for 2, 3, 4, and 5 h. Cur was labeled with Rhodamine B (shown in red), while cell nuclei were labeled with Hoechst 33,342 (shown in blue). The scale bars present 100 μm

Further studies were conducted to determine the cellular uptake of CaCO_3_@Cur@QTX125@HA nanoparticles in different cancer cells. To enhance visualization, Cur was labeled with Rhodamine B (shown in red) and loaded into the nanoparticles, while cell nuclei were stained with Hoechst 33,342 (shown in blue). As shown in Fig. 4a, after incubation with Rhodamine B-labeled CaCO_3_@Cur@QTX125@HA nanoparticles for 1 h, an obvious red fluorescence was observed in HCT 116 cells. With further increase in incubation time, the intensity of red fluorescence significantly increased. Importantly, the red fluorescence evenly distributed inside the cells, indicating that the CaCO_3_@Cur@QTX125@HA nanoparticles did not aggregate. Similarly, high cellular uptake of CaCO_3_@Cur@QTX125@HA nanoparticles was observed in HT29 cells even after only 1 h of incubation, and the efficiency of uptake maintained at a high level with increasing incubation time. This high cellular uptake efficiency may be attributed to the HA coating, which can specifically target the CD44 ligand on cancer cell surface and facilitate the cellular uptake of CaCO_3_@Cur@QTX125@HA nanoparticles (Fig. 4b). To further confirm this, the cellular uptake of CaCO_3_@Cur@QTX125@HA nanoparticles was tested in HCV-29 and UC-3 cells, and high uptake efficiency was similarly observed (Additional file 1: Figures S5, S6). These results demonstrate that CaCO_3_@Cur@QTX125@HA nanoparticles have great potential for delivering Cur into various cancer cells.

### Inhibitory effects of CaCO_3_@Cur@QTX125@HA nanoparticles on CRC cell growth

**Fig. 5:**
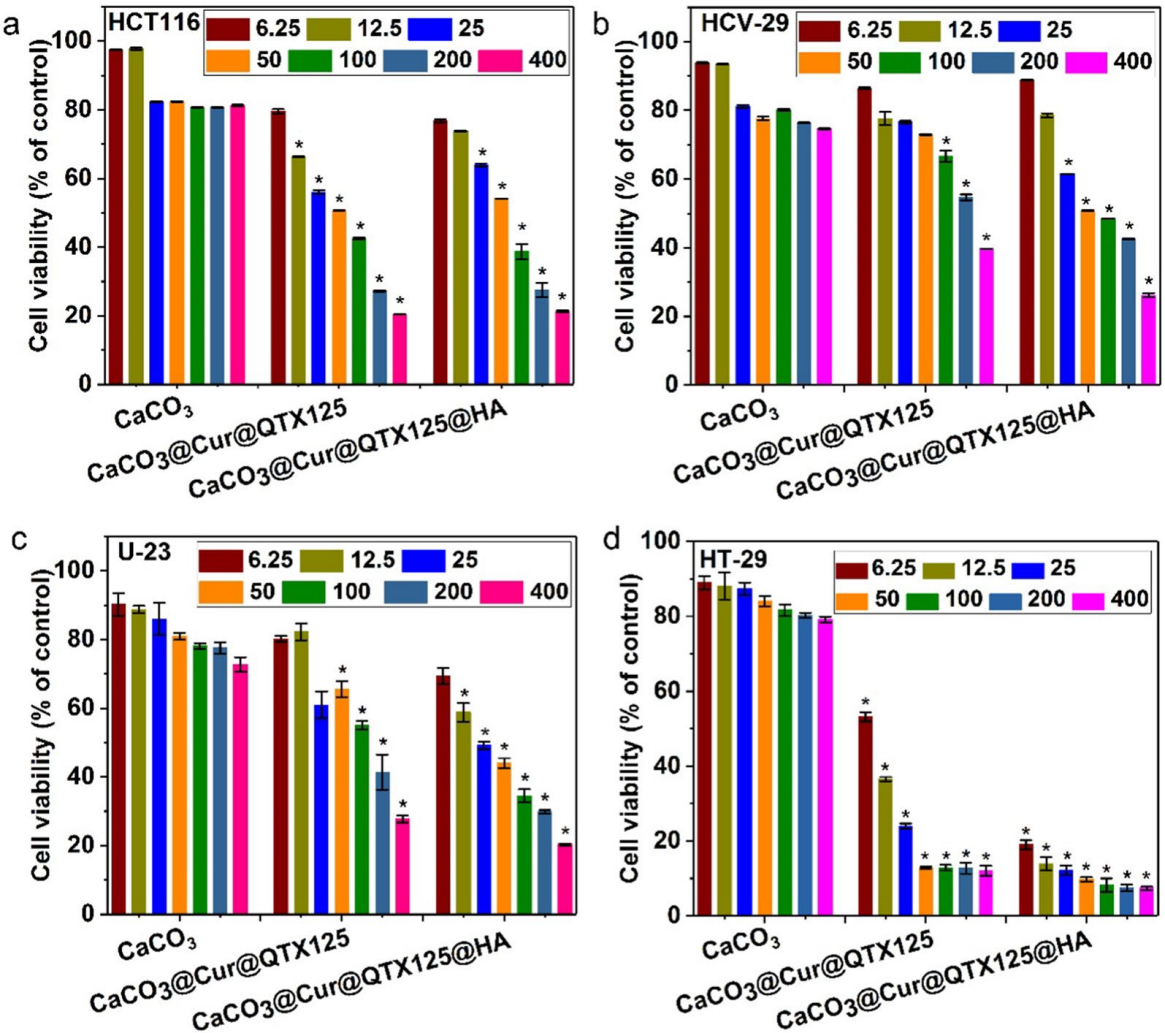
Metabolic activity of HCT116, HCV-29, U-23 and HT-29 cells after incubation with CaCO_3_, CaCO_3_@Cur@QTX125 or CaCO_3_@Cur@QTX125@HA nanoparticles with a concentration of Cur of 6.25, 12.5, 25, 50, 100, 200 or 400 μg/mL

The inhibitory effects of CaCO_3_, CaCO_3_@Cur@QTX125, and CaCO_3_@Cur@ QTX125@HA nanoparticles on the growth of various cancer cells were measured using Alamarblue assays. The cells were incubated with Cur at concentrations ranging from 6.25 μg/mL to 400 μg/mL for 48 h. As shown in Fig. 5a, in HCT116 cells, regardless of the concentration of Cur, the CaCO_3_ nanoparticles alone did not induce obvious cytotoxicity, and cell metabolic activity was preserved above 80%. In contrast, treatment with CaCO_3_@Cur@QTX125 nanoparticles led to decreased metabolic activity in a concentration-dependent manner. As the concentration of Cur increased from 6.25 μg/mL to 400 μg/mL, the metabolic activity decreased from 80 to 20%. After coating with HA, similar results were observed. In HCV-29 and U-23 cells, CaCO_3_ did not induce obvious cytotoxicity (Fig. 5b, c). However, when treated with increasing concentrations of Cur, a clear decrease in cell metabolic activity was observed. At high concentration of 400 μg/mL, the metabolic activity of these cells after incubation with CaCO_3_@Cur@QTX125 nanoparticles was only40% and 28%, respectively. Interestingly, after coating with HA, the inhibitory effects were further increased to varying degrees, indicating that HA enhanced the inhibitory effects of CaCO_3_@Cur@QTX125 nanoparticles. This enhancement was even greater in HeLa and IEC-6 cells (Additional file 1: Figures S7, S8). For example, in HeLa cells, the cell metabolic activity after incubation with CaCO_3_@Cur@QTX125@HA nanoparticles was only 15%, compared to 62% for cells treated with CaCO_3_@Cur@QTX125 counterparts. Similarly, the cell metabolic activity of IEC-6 cells decreased from 50 to 36% after treatment with CaCO_3_@Cur@QTX125@HA nanoparticles at a Cur concentration of 200 μg/mL. In contrast to the above cancer cells, significantly stronger inhibitory effects were observed in HT-29 cells, with much lower cell metabolic activity (54% and 36%) at Cur concentrations of 6.25 μg/mL and 12.5 μg/mL when treated with CaCO_3_@Cur@QTX125 nanoparticles. Moreover, the inhibitory effects of were CaCO_3_@Cur@QTX125 nanoparticles further enhanced after coating with HA (54% vs 14%). Importantly, as the Cur concentration increased to 25 μg/mL, 50 μg/mL, 100 μg/mL, 200 μg/mL, and 400 μg/mL, the cell metabolic activity was as low as < 12% and 7% after treatment with CaCO_3_@Cur@QTX125 and CaCO_3_@Cur@QTX125@HA nanoparticles, demonstrating that both of these nanoparticles have high inhibitory effects on the growth of cancer cells, indicating the good synergistic effects of Cur and QTX125. Encouragingly, CaCO_3_@Cur@QTX125 and CaCO_3_@Cur@QTX125@HA nanoparticles exhibit obvious specific inhibitory effects towards CRC cells, highlighting their potential application for the treatment of CRC.

### Evaluation of therapeutic effects of CaCO_3_@Cur@QTX125@HA nanoparticles in PDO models

**Fig. 6:**
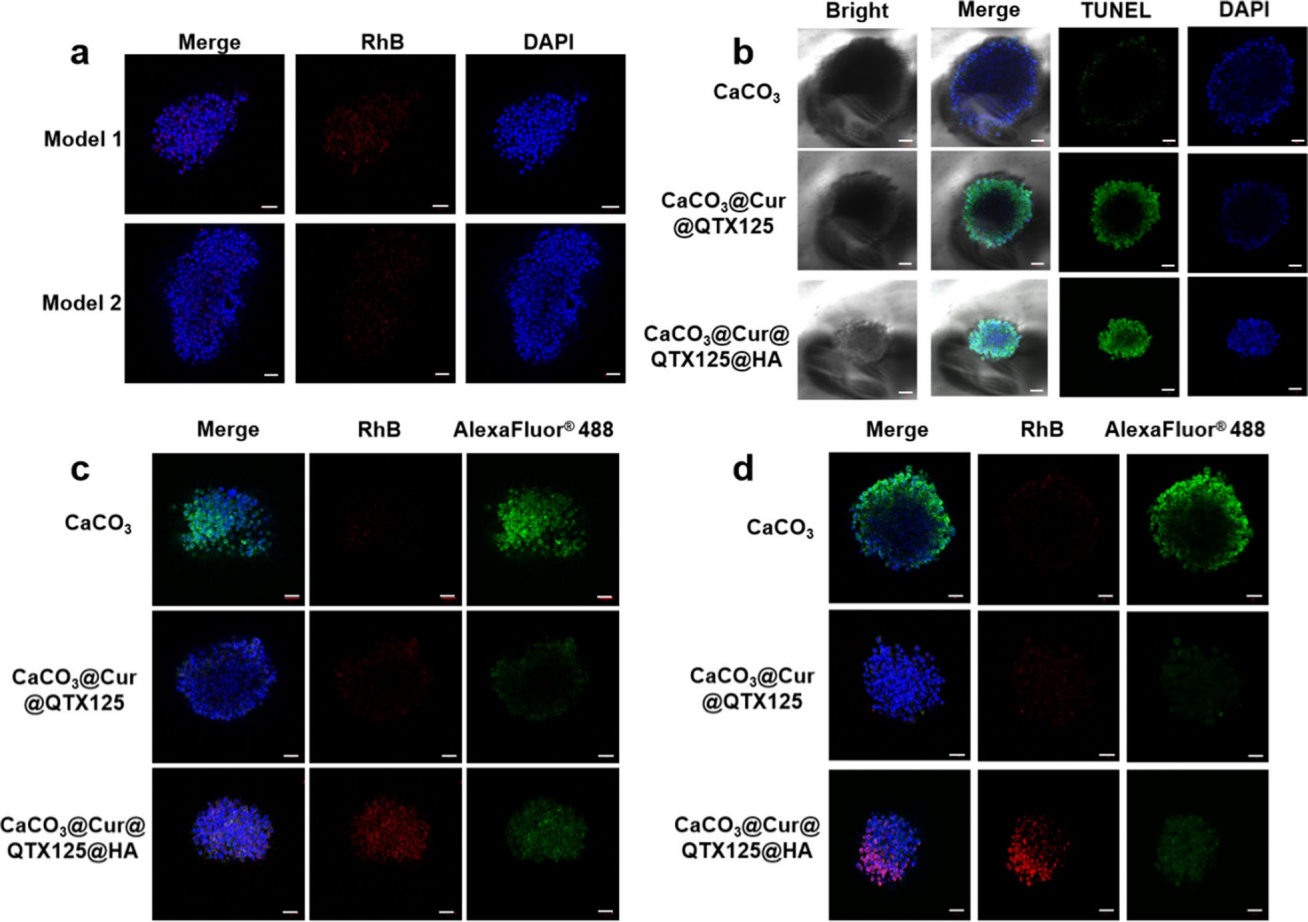
**a** CaCO_3_@Cur@QTX125@HA Image of the Z-stack in the PDO model of colorectal cancer. Blue: DAPI-labeled nucleus, red: nanoparticles labeled with RhB). Scale bar: 50 μ m; **b** Representative confocal images of PDO models after treatment with CaCO_3_, CaCO_3_@Cur@QTX125 and CaCO_3_@Cur@QTX125@HA nanoparticles, measured by TUNEL assay. **c** Expression of tumor marker CEA in CRC PDO model after incubation with CaCO_3_, CaCO_3_@Cur@QTX125 and CaCO_3_@Cur@QTX125 @HA nanoparticles at a Cur concentration of 20 μg/mL; **d** Expression of tumor marker CA19-9 in CRC PDO model after incubation with CaCO_3_, CaCO_3_@Cur@QTX125 and CaCO_3_@Cur@QTX125@HA nanoparticles at a Cur concentration of 20 μg/mL. The nuclei were labeled with DAPI in blue, Cur were labeled with Rhodamine B in red, and tumor markers were labeled with AlexaFluor^®^ 488 in green. The scale bars represent 50 μm

Organoids are a simple yet important approach for evaluating the therapeutic effect of CRC in vitro. Therefore, to evaluate the effectiveness of CaCO_3_@Cur@QTX125@HA nanoparticles on CRC, two patient derived colorectal carcinoid organ (Colon PDO) models were constructed in vitro (Additional file 1: Figure S9). The uptake of CaCO_3_@Cur@QTX125@HA nanoparticles in the organoid was first determined using Z-stack confocal microscopy. The red fluorescence from Rhodamine B labeled Cur was clearly observed near the nucleus stained with DAPI (blue fluorescence), demonstrating the excellent internalization effect of CaCO_3_@Cur@QTX125@HA nanoparticles in PDO (Fig. 6a). In comparison to other CaCO_3_-based nanoparticles, CaCO_3_@Cur@QTX125@HA exhibited superior internalization in Colon PDO models (Fig. 6c). Next, cell apoptosis in CRC PDO after incubation with CaCO_3_, CaCO_3_@Cur@QTX125, and CaCO_3_@Cur@QTX125@HA nanoparticles was determined using TUNEL assay (Fig. 6b), where fluorescence intensity reflects the level of apoptosis. It was observed that the green fluorescence was lowest in CRC PDO treated with CaCO_3_ nanoparticles, indicating their low cytotoxicity. After 12 days of treatment, PDOs incubated with CaCO_3_@Cur@QTX125@HA nanoparticles exhibited more obvious cell apoptosis, possibly due to HA’s ability to enhance cellular uptake in PDO models (Additional file 1: Figure S10). Immunofluorescence was used to detect tumor marker expression in PDO models. Red fluorescence intensity was used to measure nanoparticle internalization in PDO models, while green fluorescence was utilized to indicate higher CEA and CA19-9 expression on the cell surface (Fig. 6c, d). After treatment with CaCO_3_ nanoparticles, the PDO model exhibited the strongest green fluorescence, indicating higher expression of CEA and CA19-9 on the cell surface. In contrast, the green fluorescence was fainter in the PDO models treated with CaCO_3_@Cur@QTX125 and CaCO_3_@Cur@QTX125@HA nanoparticles, possibly due to the significant pro-apoptotic effects of CaCO_3_ nanoparticles in PDO cells. Bax protein expression was further measured by immunofluorescence assay to determine the effect of different nanoparticles on apoptosis in the PDO models. It was observed that compared to treatment with CaCO_3_ nanoparticles, Bax protein expression significantly increased in PDOs treated with CaCO_3_@Cur@QTX125 and CaCO_3_@Cur@QTX125@HA nanoparticles, with the latter demonstrating the most obvious fluorescent intensity (Fig. 6d). These results indicate that CaCO_3_@Cur@QTX125@HA nanoparticles have a significant inhibitory effect on the PDO model of CRC.

## Conclusions

Multifunctional CaCO_3_@Cur@QTX125@HA nanoparticles were prepared using a one-pot gas diffusion strategy and were used to inhibit the growth of colorectal cancer (CRC) cells. The amorphous CaCO_3_@Cur@QTX125@HA nanoparticles exhibited uniform, spherical, and hierarchical morphology with a diameter of approximately 450 nm and a Zeta potential of − 8.11 mV. Cur was released from the CaCO_3_@Cur@QTX125@HA over 5 h upon nanoparticle degradation. The CaCO_3_@Cur@QTX125@HA nanoparticles showed high cellular uptake efficiency in multiple cancer cells, resulting in an effective inhibition of cancer cell growth, especially in CRC cells. Moreover, these nanoparticles were effectively taken up by PDO models, inducing cancer cell apoptosis. These results highlight the potential of CaCO_3_@Cur@QTX125@HA nanoparticles for CRC treatment.

### Supplementary Information

**Additional file 1: Figure S1.** SEM image of representative CaCO_3_ nanoparticles. The scale bar presents 10 μm. **Figure S2.** SEM image of representative CaCO_3_@Cur@QTX125 nanoparticles. The scale bar presents 20 μm. **Figure S3.** SEM image of representative CaCO_3_@Cur@QTX125@HA nanoparticles. The scale bar presents 50 μm. **Figure S4.** Elemental mapping of CaCO_3_@Cur@QTX125 nanoparticles, scale bars represent 5 μm. **Figure S5.** Fluorescence images of HCV-29 cells after incubation with CaCO_3_@Cur@QTX125@HA for 2, 3, 4, and 5 h, scale bars represent 100 μm. **Figure S6.** Fluorescence images of HCV-29 cells after incubation with CaCO_3_@Cur@QTX125@HA for 2, 3, 4, and 5 h, scale bars represent 100 μm. **Figure S7.** Metabolic activity of HeLa cells after incubation with CaCO_3_, CaCO_3_@Cur@QTX125 or CaCO_3_@Cur@QTX125@HA nanoparticles with a concentration of Cur of 12.5, 25, 50, 100 or 200 μg/mL. **Figure S8.** Metabolic activity of IEC-6 cells after incubation with CaCO_3_, CaCO_3_@Cur@QTX125 or CaCO_3_@Cur@QTX125@HA nanoparticles with a concentration of Cur of 12.5, 25, 50, 100 or 200 μg/mL. **Figure S9.** Representative morphological image of PDO models with different CRC1/2 (PDO1/2). The scale bars represent 100 μm. **Figure S10.** Images of the growth morphology of the PDO1/2 model at Day 1, 3, 5, 9 and 12 days. The number of organoids per well was counted at the end of the experiment. The scale bars represent 100 μm.

## Data Availability

Not applicable.
