# Supplementary material for: Multifunctional CaCO3@Cur@QTX125@HA nanoparticles for effectively inhibiting growth of colorectal cancer cells
Source: J Nanobiotechnology. 2023 Sep 29;21:353. doi: 10.1186/s12951-023-02104-w (PMC10543835; doi:10.1186/s12951-023-02104-w)
Supplement: Supplementary file 1 — Additional file 1: Figure S1. SEM image of representative CaCO3 nanoparticles. The scale bar presents 10 μm. Figure S2. SEM image of representative CaCO3@Cur@QTX125 nanoparticles. The scale bar presents 20 μm. Figure S3. SEM image of representative CaCO3@Cur@QTX125@HA nanoparticles. The scale bar presents 50 μm. Figure S4. Elemental mapping of CaCO3@Cur@QTX125 nanoparticles, scale bars represent 5 μm. Figure S5. Fluorescence images of HCV-29 cells after incubation with CaCO3@Cur@QTX125@HA for 2, 3, 4, and 5 h, scale bars represent 100 μm. Figure S6. Fluorescence images of HCV-29 cells after incubation with CaCO3@Cur@QTX125@HA for 2, 3, 4, and 5 h, scale bars represent 100 μm. Figure S7. Metabolic activity of HeLa cells after incubation with CaCO3, CaCO3@Cur@QTX125 or CaCO3@Cur@QTX125@HA nanoparticles with a concentration of Cur of 12.5, 25, 50, 100 or 200 μg/mL. Figure S8. Metabolic activity of IEC-6 cells after incubation with CaCO3, CaCO3@Cur@QTX125 or CaCO3@Cur@QTX125@HA nanoparticles with a concentration of Cur of 12.5, 25, 50, 100 or 200 μg/mL. Figure S9. Representative morphological image of PDO models with different CRC1/2 (PDO1/2). The scale bars represent 100 μm. Figure S10. Images of the growth morphology of the PDO1/2 model at Day 1, 3, 5, 9 and 12 days. The number of organoids per well was counted at the end of the experiment. The scale bars represent 100 μm. [file 12951_2023_2104_MOESM1_ESM.docx]

**Additional file Information**

**Multifunctional CaCO_3_@Cur@QTX125@HA Nanoparticles for Effectively Inhibiting Growth of Colorectal Cancer Cells**

Shengyun Hu^1,†^, Kunkun Xia^1,†^, Xiaobei Huang^5,†^, Ye Zhao^2^, Qingqing Zhang^1^, Dongdong Huang^3^, Weiyi Xu^4^, Zhengju Chen^3*^, Chenfei Wang^4*^, Zhiyong Zhang^1*^

^1^Department of Colorectal and Anal Surgery, the First Affiliated Hospital of Zhengzhou University, Zhengzhou 450052, China

^2^Department of Gastroenterology, The First Affiliated Hospital of Zhengzhou University, Zhengzhou 450052, China

^3^Pooling Medical Research Institutes of 100Biotech, Beijing 100006, China

^4^Department of Dermatology, Children’s Hospital of Fudan University, National Children's Medical Center, Shanghai, 201102, China

^5^Chongqing Institute of Green and Intelligent Technology, Chinese Academy of Sciences, Chongqing 400714, China

^†^These authors contributed to this work equally.

Email: [fcczhangzy2@zzu.edu.cn](mailto:fcczhangzy2@zzu.edu.cn) (Z. Zhang); melody@100biotech.com (Z. Chen); [wangchenfei@fudan.edu.cn](mailto:wangchenfei@fudan.edu.cn) (C. Wang)


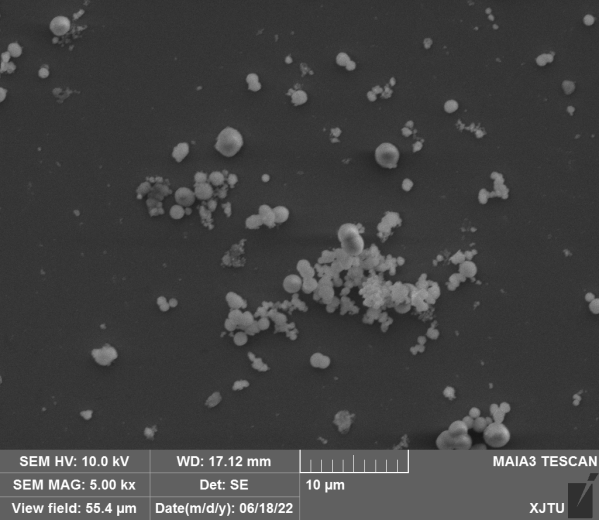


**Figure S1.** SEM image of representative CaCO_3_ nanoparticles. The scale bar presents 10 μm.


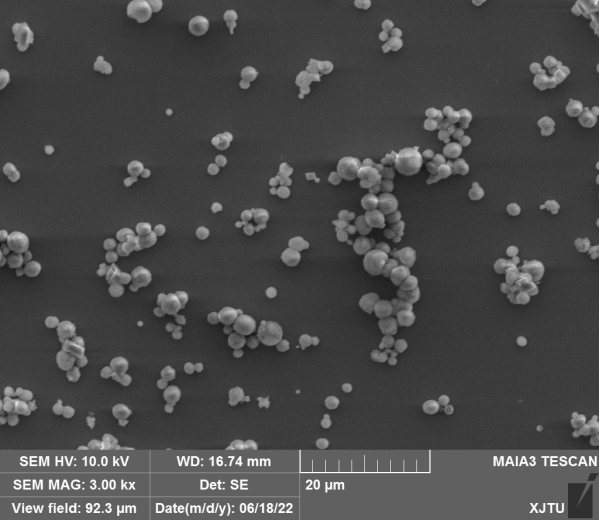


**Figure S2.** SEM image of representative CaCO_3_@Cur@QTX125 nanoparticles. The scale bar presents 20 μm.


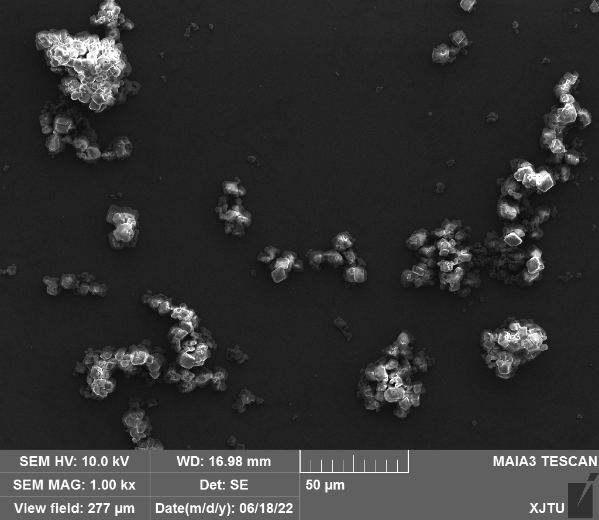


**Figure S3.** SEM image of representative CaCO_3_@Cur@QTX125@HA nanoparticles. The scale bar presents 50 μm.


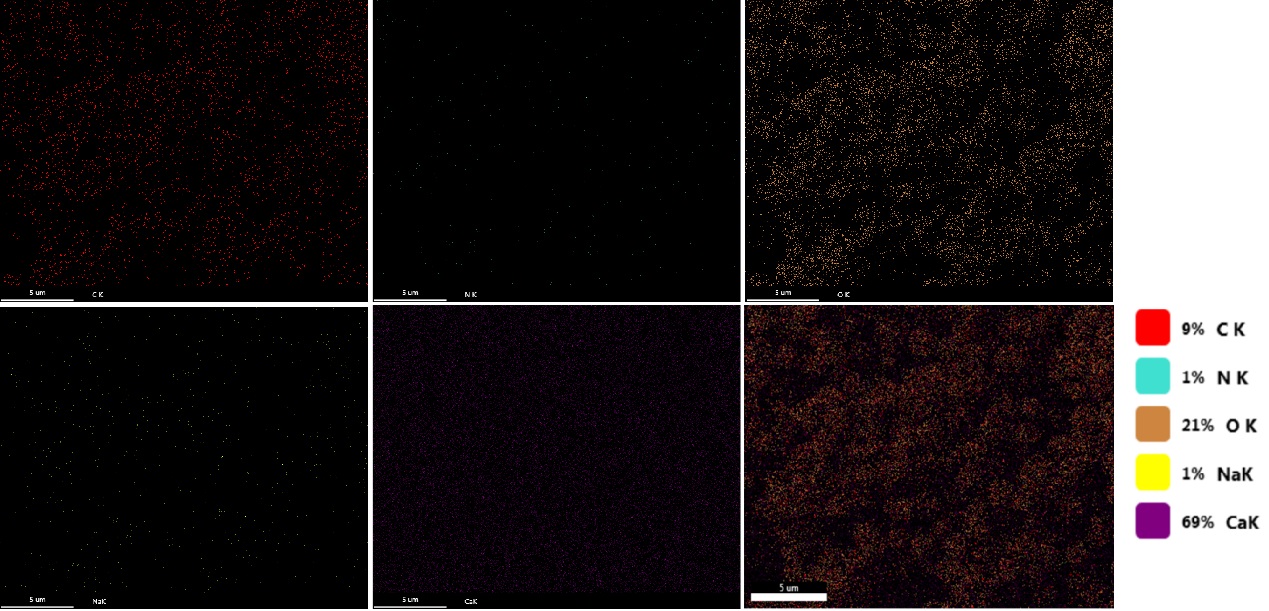


**Figure S4.** Elemental mapping of CaCO_3_@Cur@QTX125 nanoparticles, scale bars represent 5 μm.


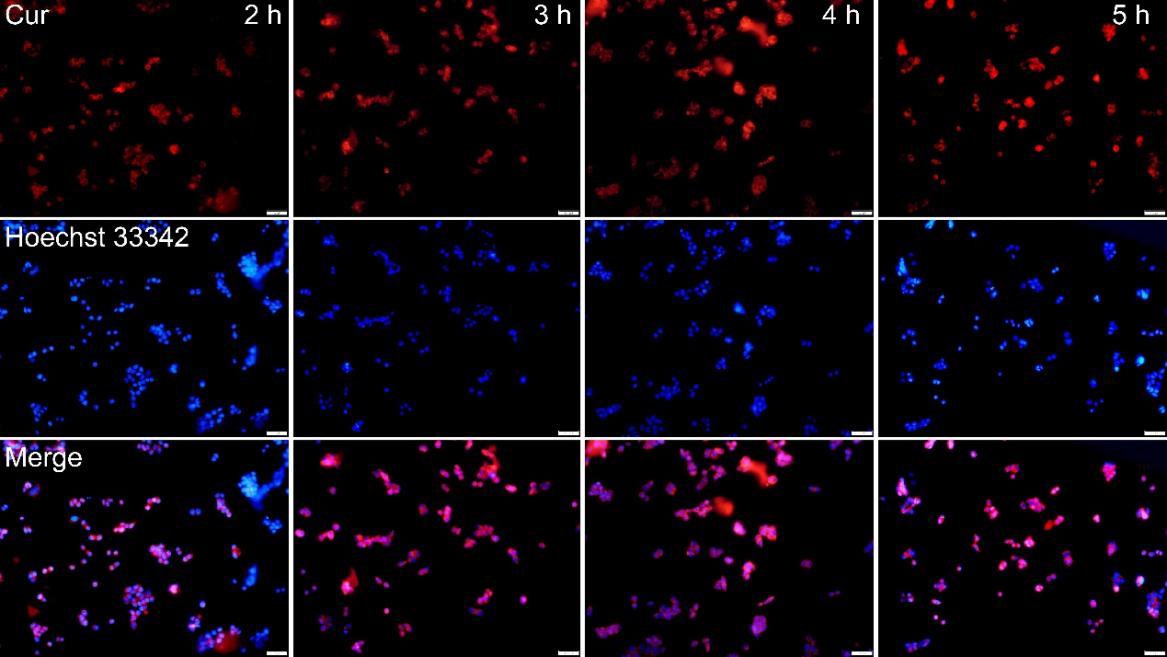


**Figure S5.** Fluorescence images of HCV-29 cells after incubation with CaCO_3_@Cur@QTX125@HA for 2, 3, 4, and 5 hours, scale bars represent 100 μm.


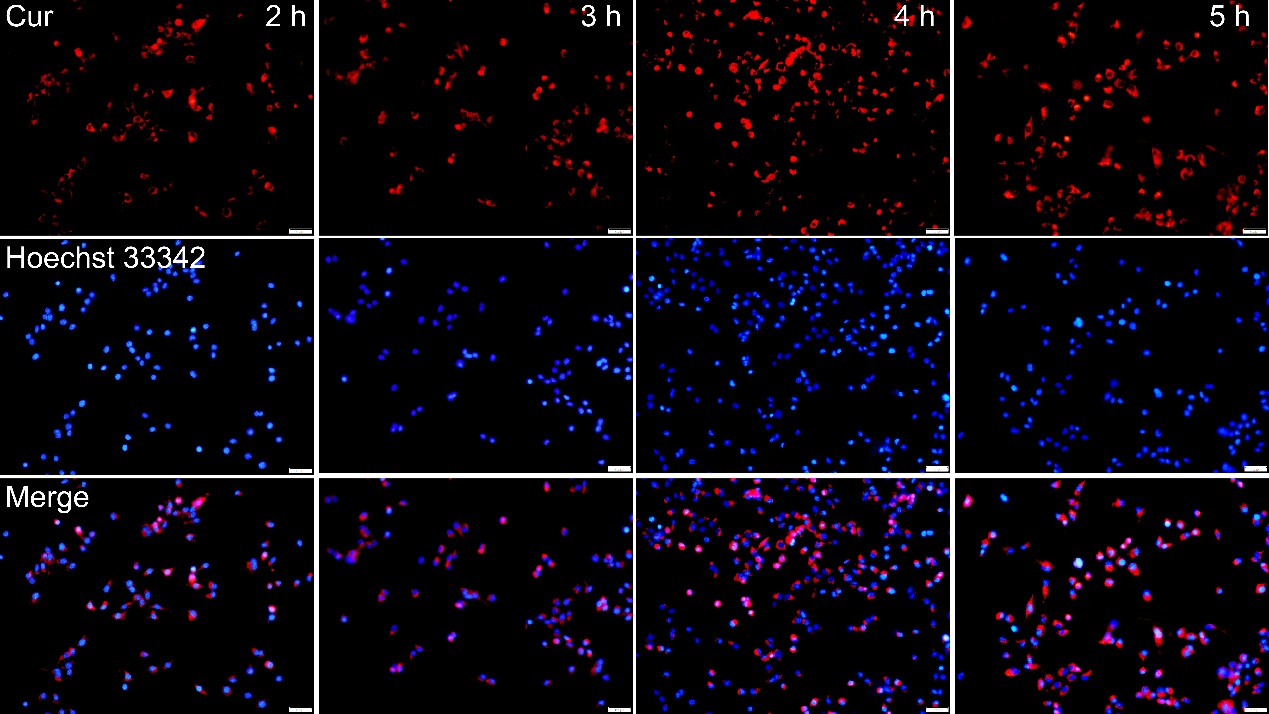


**Figure S6.** Fluorescence images of HCV-29 cells after incubation with CaCO_3_@Cur@QTX125@HA for 2, 3, 4, and 5 hours, scale bars represent 100 μm.





**Figure S7.** Metabolic activity of HeLa cells after incubation with CaCO_3_, CaCO_3_@Cur@QTX125 or CaCO_3_@Cur@QTX125@HA nanoparticles with a concentration of Cur of 12.5, 25, 50, 100 or 200 μg/mL.





**Figure S8.** Metabolic activity of IEC-6 cells after incubation with CaCO_3_, CaCO_3_@Cur@QTX125 or CaCO_3_@Cur@QTX125@HA nanoparticles with a concentration of Cur of 12.5, 25, 50, 100 or 200 μg/mL.

**Figure S9.** Representative morphological image of PDO models with different CRC1/2 (PDO1/2). The scale bars represent 100 μm.

**Figure S10.** Images of the growth morphology of the PDO1/2 model at Day1, 3, 5, 9 and 12 d. The number of organoids per well was counted at the end of the experiment. The scale bars represent 100 μm.
